# Detection of *H. pylori* Infection in Atherosclerotic Plaques of 180 Corpses in Referred to Forensic Medicine Center of Tehran in 2016–2017

**Published:** 2018-11

**Authors:** Jaber GHAREHDAGHI, Reza NASR, Jamil KHEIRVARI KHEZERLOO, Mohammad Reza AKBARI EIDGAHI, Mohsen TABASI, Mohammadreza GHASEMI, Elnaz MOZAFFARI, Reza AZIZIAN, Mohammad Reza ESKANDARION

**Affiliations:** 1. Legal Medicine Research Center, Legal Medicine Organization, Tehran, Iran; 2. Semnan Biotechnology Research Center, Semnan University of Medical Sciences, Semnan, Iran; 3. Dept. of Molecular Biology, Pasteur Institute of Iran, Tehran, Iran; 4. Dept. of Biology, Damghan Branch, Islamic Azad University, Damghan, Iran; 5. Massoud Clinical Laboratory, Tehran, Iran

## Dear Editor-in-Chief

Atherosclerosis is a major cause of cardiovascular disease, and infections such as *Helicobacter pylori* infection are associated with atherosclerosis (1). The aim of this study was to determine the relationship between *H. pylori* infection and atherosclerosis. Many studies have confirmed the possibility of atherosclerosis by infection, but bacteria like *H. pylori* and *Chlamydia pneumoniae* and viruses such as *Cytomegalovirus* implicated in atherosclerosis.

In this cross-sectional study, 180 samples including 90 samples from the corpses which had atherosclerotic plaque artery (60 ischemic and 30 abdominal aorta) and 90 samples from the corpses did not have any plaque of atherosclerosis, or clogged arteries (60 ischemic and 30 abdominal aorta) were assayed by PCR and analyzed by Chi-square test (2).

**Fig. 1:**
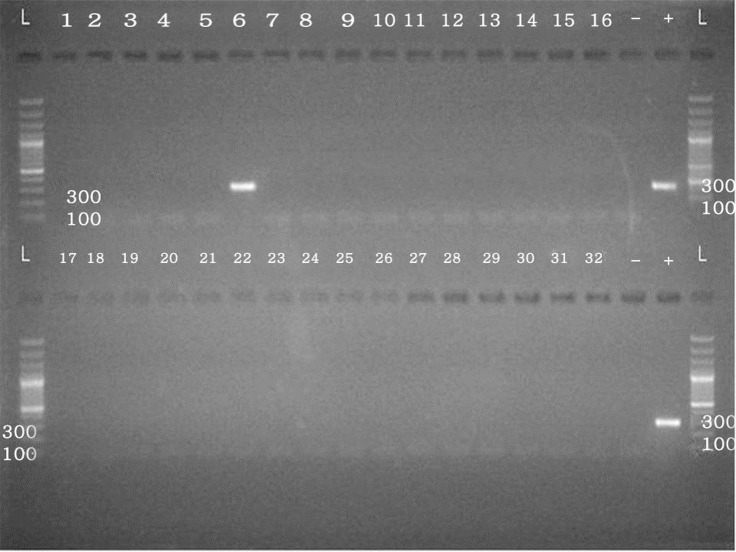
DNA genomic of *H. pylori* in atherosclerosis plaque. Band 6 shows the positive sample and the others show negative samples. Size marker (Fermentas) with product number 0321 SM on the right side of the gel and the band size is specified at the top of each. Bands are in the expected size range

Ten percent of samples taken from plaques were positive for *H. pylori* DNA, 6.6% were in coronary arteries and 3.3% were in aortic wall samples. None of the samples of the control group contained *H. pylori* bacteria (Fig. 1). Bands of electrophoresis with size of 200–300 bp in the PCR product were considered as a positive result. This band has shown the amplification fragment of the gene from the bacterium *H. pylori*. There was no statistically significant difference between the two groups in terms of history of specific diseases such as blood hypertension, hyperlipidemia, smoking and diabetes (Table 1).

**Table 1:** Comparing the percentage of various variants in the two studied groups

***Risk factor***	***Corpses with atherosclerotic plaques (%)***	***Corpses without atherosclerotic plaques (%)***	***P-value***
Blood hypertension	27 (30)	24 (28)	> 0.05
Hyperlipidemia	34 (38)	31 (35)	> 0.05
Smoking	31 (35)	27 (30)	> 0.05
Diabetes	14 (20)	9 (16)	> 0.05

The high cost of cardiovascular diseases on the economy and society, and understanding the pathophysiology of atherosclerosis, are very useful in treatment and further researches. Increased levels of pro-inflammatory cytokines and lipid profile with an increased risk of cardiovascular events are associated (3).

There was a significant correlation between identification of *H. pylori* genome and coronary artery disease. Therefore, this infection is included in the list of criteria for treatment as a risk factor for related groups. The role of *H. pylori* in the pathogenesis of atherosclerosis was shown in some study but was not found a significant relationship between cardiovascular risk factors with these organisms (4). There is only limited relation between *H. pylori* infection and cardiovascular disease, but these researches did not reject any association between this infection and cardiovascular disease, more epidemiological studies needed for confirmation of this idea. 14.3% of patients with atherosclerosis were positive for *H. pylori* DNA, which again consistent with these results (5). However, in some of these studies in contrast to results of our study, there was no significant association.

In line of this argumentation, 85 patients with atherosclerosis were performed by PCR. *H. pylori* DNA in patients with atherosclerotic plaque were 4.7% and internal thoracic artery biopsy had 3.5%; therefore, was not found any significant difference between the two groups (6). Reasons for obtaining such variable results by PCR are suggested to include patchy distribution of *H. pylori* in atherosclerotic lesions, insufficient atheromatous tissue examined lack of standardization of laboratory methods, inability to perform *H .pylori* DNA extraction, temporary colonization of *H. pylori* in a particular place of the tissues and the presence of PCR inhibitors in atheroma plaques. In addition, this may interfere with certain strains of microbes in cardiovascular events; this study was not carried out to differentiate strains. Another reason can use various methods to identify infections or studies in different populations and different geographic areas. Nevertheless, more comprehensive studies with considering the other factors such as age, type of antimicrobial, the economic and social situation, various origin tissues and geographical regions needed.
